# Machine learning techniques for classifying dangerous asteroids

**DOI:** 10.1016/j.mex.2023.102337

**Published:** 2023-08-19

**Authors:** Seyed Matin Malakouti, Mohammad Bagher Menhaj, Amir Abolfazl Suratgar

**Affiliations:** Distributed and Intelligent Optimization Research Laboratory, Department of Electrical Engineering, Amirkabir University of Technology, Tehran, Iran

**Keywords:** Machine learning, Asteroids, NASA, Hazards of asteroids, Machine learning

## Abstract

There is an infinite number of objects in outer space, and these objects and asteroids might be harmful. Hence, it is wise to know what is surrounding us and what can harm us amongst those.

Therefore, in this article, with the hyperparameters tuning of Extra Tree, Random Forest, Light Gradient Boosting Machine, Gradient Boosting, and Ada Boost, the hazards of asteroids around the Earth were classified, and the results of ROC Curves for these algorithms were compared.

• Reviewing the list of NASA-certified asteroids classified as the nearest Earth object
• Investigating the risk of asteroids with the help of Extra Tree, Random Forest, Light Gradient Boosting Machine, Gradient Boosting, and Ada Boost
• Comparing the performance of machine learning algorithms in the classification of high-risk asteroids

## Graphical abstract

**Figure:**
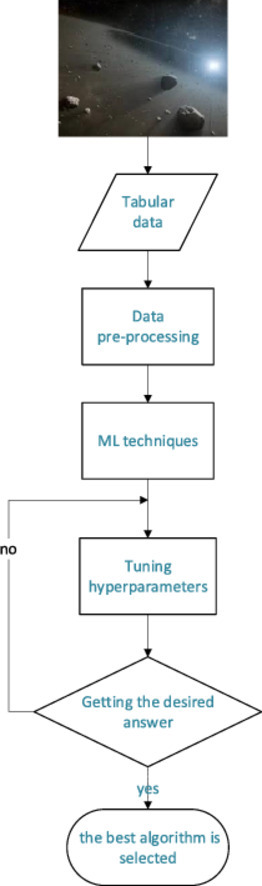

Specifications table

**Table:** 

Subject area	Physics and Astronomy
More specific subject area	Dangerous Asteroids
Name of your method	Machine learning
Name and reference of the original method	Extra Tree, Random Forest, Light Gradient Boosting Machine, Gradient Boosting, and Ada Boost
Resource availability	https://www.kaggle.com/datasets/shrutimehta/nasa-asteroids-classification

## Method details

### Extra tree (ET) and random forest (RF)

RF [1] employs bootstrap copies for testing. However, the input data is replaced with subsamples rather than the original sample. Utilizing the complete input samples in the Extra Trees sklearn implementation to bootstrap replicas. Since bootstrapping makes it more varied, this might lead to a higher degree of uncertainty.

Another distinction is to choose cut points for nodes to be broken up. TRandom Forest selects the optimal split while Extra Trees [2] randomly picks it. When the split points are chosen, these two algorithms decide which of the subsets of features is better. Extra Trees, on the other hand, introduce randomness while maintaining optimization.

Bias and variance reductions are motivated by these differences. Alternatively, preference may be reduced by utilizing the original sample rather than a bootstrap copy. Each node's breakpoint may be chosen at random to minimize volatility. The Extra Trees method is more efficient in processing resources and execution time. Since every step is the same, this technique saves time by not having to determine the ideal split point. Extra Tree gets its name from all of these factors.

An RF is a group of i_tree indicators $g(z;t_{k})$k=1,...,K*,* where *z* represents the measured input vector of length ρ accompanied by a random vector z. The $\beta_{k}$ give below are random, independent, and independently distributed vectors. The assumption is that the training data will be separately derived from the joint distribution of (Z,C) which *C* is numerical outcome and *n* is the number of Extra tree’s prediction and comprises n(ρ+1) and tuples $\left(z_{1},c_{1}\right),...,\left(z_{n},c_{n}\right)$.

The unweighted average throughout the sample is the random forest forecast:

$$\bar{g}\left(z\right)=\left(1/k\right)\sum_{k=1}^{K}g\left(z;\beta_{k}\right).$$

As k→∞ the law of Large Numbers says:

(1) $$E_{X,Y}{\left(C-\bar{g}\left(z\right)\right)}^{2}\to E_{Z,C}{\left(C-E_{\beta }g\left(Z;\beta \right)\right)}^{2}$$

The amount on the right represents the RF's prediction error $PE_{f}^{*}$. Since formula 1 is convergent, it follows that RF does not overfit. Therefore, this algorithm quantify the typical inaccuracy g(Z;β) in our predictions for a given tree as:

(2) $$PE_{t}^{*}=E_{\beta }E_{ZC}{\left(C-g\left(Z;\beta \right)\right)}^{2}$$

## Light GBM

It is used for decision tree-based machine learning tasks such as classification, ranking, etc. There are three reasons behind the speed of Light GBM [3]:

• histogram-based segmentation [4]
• one-Side Sampling Using Gradient (GOSS) [5]
• Unique Function Bundling (EFB) [6]

## Gradient boosting

A machine learning method called gradient boosting is used, among other things, for classification and regression problems. It provides a prediction model as an ensemble of decision trees like weak prediction models.

When should one utilize gradient boosting [7]? The answer is given as below:

i) The Gradient Boosting Algorithm is often used to reduce bias error.
ii) The gradient boosting algorithm may solve classification and regression issues. MSE is the cost function for regression issues, whereas Log-Loss is the function for classification issues.

## Ada Boost

AdaBoost classifiers [8] are meta-estimators that begin by fitting a classifier to the original dataset. It then applies more copies of the classifier to the same dataset but it modifies the weights of poorly categorized examples such that subsequent classifiers focus more on challenging situations.

## Case study

Natural objects with orbits into the inner solar system have often struck our planet since its creation (more than 4.5 billion years ago). These objects are typically referred to as Near-Earth Objects (NEAs) [9], which are likely leftover pieces of Main-Belt Asteroids that evolved after collisional events until reaching an orbit that approaches Earth [[10], [11]]. The first Near-Earth Object, 433 Eros, was founded in 1898, but interest in Near-Earth Objects grew during the Apollo program when it was shown that lunar craters are the result of collisions [12,13]. In many instances, the limited number of observations makes estimating the orbit of a tiny natural body difficult. Even if a preliminary orbit is known, the uncertainty is considerable. The only continuation option is to evaluate a collection of orbits corresponding to a confidence zone with acceptable astrometric residuals [14]. These orbits, created by sampling (randomly or geometrically), are known as Virtual Asteroids (VAs). The objective of Collision Monitoring (IM) is to determine if the CR includes a Virtual Impactor (VI), a subset of beginning circumstances that, when propagated, indicates an impact with the Earth [15]. in this situation, a VI is a connected component. Once a representative of a VI (the beginning circumstances that lead to a collision) has been found, the Impact Probability (IP) of the VI must be computed. In general, the IP of a VI is proportional to its volume in the space of the orbital elements. If we seek remote IPs, the sampling should be pretty dense; nevertheless, determining how to achieve this while ensuring the completeness of VI searches while accounting for processing costs is difficult. One family of approaches, which includes Monte Carlo (MC) and ranging statistical methods [16,17], studies the probabilistic distributions of the orbits across the swarm of Virtual Asteroids using random sampling of the CR. When we need to handle a vast library of items and modest probabilities with a few Virtual Asteroids, sampling the CR with a geometrical object, such as a smooth manifold, is more cost-effective.

In this article, it was tried to improve the performance of Extra Tree (ET) and Random Forest (RF), Light GBM, Gradient Boosting and Ada Boost algorithms in the Dangerous Asteroids classification using the Grid Search CV method.

## Data

Asteroids that can arrive in proximity or collide with the Earth are hazardous asteroids. Therefore, it would be analyzed large datasets to identify all the possibly dangerous asteroids by researchers [18,19], so in this manuscript NASA data was used to Classify Dangerous Asteroids with ML methods. data collected by NASA, including 90,836 rows and ten columns representing the data's characteristics, were analyzed, and the data have some features. Names of features:

Id: Unique Identifier for each Asteroid, Name: Name given by NASA, Absolute Magnitude, Est Dia in KM(min), Est Dia in KM(max), Est Dia in M(min), Est Dia in M(max), Est Dia in Miles(min), Est Dia in Miles(max), Est Dia in Feet(min), Est Dia in Feet(max), Close Approach Date, Epoch Date Close Approach, Relative Velocity km per *sec*, Relative Velocity km per hr, Miles per hour, Miss Dist.(Astronomical), Miss Dist.(lunar), Miss Dist.(kilometers), Miss Dist.(miles), Orbiting Body, Orbit ID, Orbit Determination Date, Orbit Uncertainty, Minimum Orbit Intersection, Jupiter Tisserand Invariant, Epoch Osculation, Eccentricity, Semi Major Axis, Inclination, Asc Node Longitude, Orbital Period, Perihelion Distance, Perihelion Arg, Aphelion Dist, Perihelion Time, Mean Anomaly, Mean Motion, Equinox, Hazardous.

**Fig. 1:**
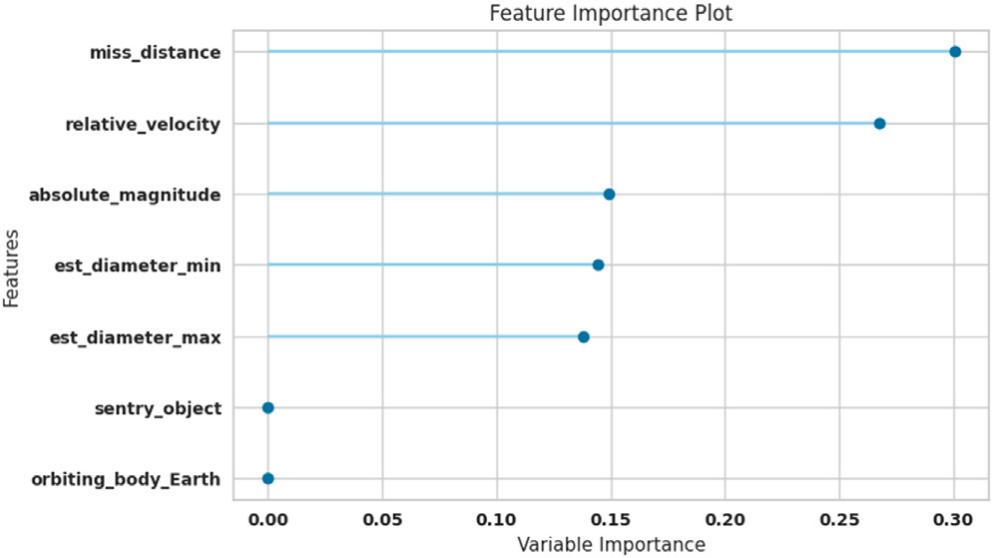
Feature importance for the classification of hazardous objects (Asteroids).

PCA method was used to reduce the dimensions of features and find the importance of the data features obtained. Fig. 1 shows that miss_distance, relative_velocity, absolute_magnitude, est_diameter_min, est_diameter_max were the most important feature among the features which have been mentioned above.

The description of the most important feature is as follows:

est_diameter_min: Minimum Estimated Diameter in Kilometres
est_diameter_max: Maximum Estimated Diameter in Kilometres
relative_velocity: Velocity Relative to Earth
miss_distance: Distance in Kilometres missed
orbiting_body: Planet that the asteroid orbits
sentry_object: Included in sentry - an automated collision monitoring system
absolute_magnitude: Describes intrinsic luminosity

Hazardous: Boolean feature that shows whether an asteroid is harmful or not. The characterization of "hazardous" depends only on whether the asteroid collided or not with the Earth.

These data were obtained at the closest approach to the Earth along a numerical integration of the orbit. The integration started from 1990 until 2022.

The integration was achieved using the name reported by NASA.

## Tuning hyperparameters

Eighty percent of the data is used in the 10-fold cross-validation and the whole of the remaining 20% of your data is unseen to the model (as the test set and validation set) [[20], [21], [22], [23], [24], [25], [26], [27], [28], [29], [30], [31]] to test the model and another portion to test it. The prediction error from Equation One is then estimated using cross-validation as follows:

(3) $$CV\left(h\right)=\frac{1}{m}\sum_{i=1}^{m}L\left(y_{i},h^{-S\left(i\right)}\left(xi\right)\right)$$

S: the number of subgroups m: the amount of the dataset

L: the loss function

$h^{-S(i)}$: the fitted function

**Fig. 2:**
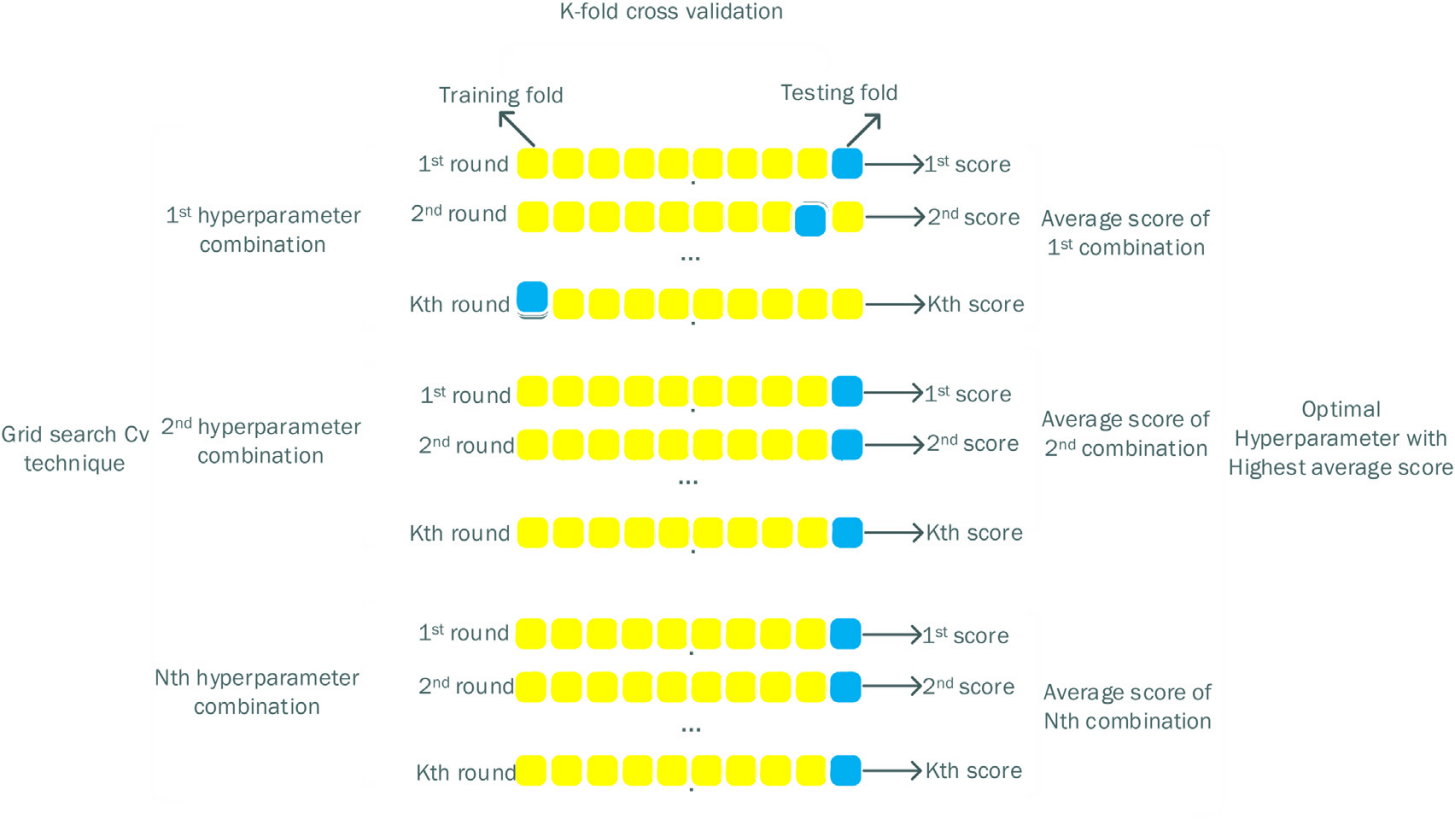
Grid search cv method.

The Grid Search CV is illustrated in Fig. 2.

## Evaluation process

The ML methods which have been brought in this manuscript can only regard the data from objects that have been detected by NASA.

Using machine learning algorithms, the “Id” and “Name” columns were removed from the data because they were added to other data by NASA to name and identify asteroids because Id and Name columns do not play a role in determining the risk of asteroids hitting the Earth.

In the first step, the outlier data were removed by the stdev() module in Python and the data were normalized by the Min-Max method. Then, the data were analyzed with the help of algorithms, the algorithms' hyperparameters were settled, and finally, Results and Discussion were discussed.

It was necessary to research machine learning methods that can analyze the data recorded by NASA to distinguish dangerous asteroids from non-dangerous ones. Hence, the grid-search CV method was used to improve the performance of Extra Tree (ET), Random Forest (RF), Light GBM, Gradient Boosting and Ada Boost algorithms.

The following metrics have been used for the analysis of celestial bodies:

**ROC** (Receiver Operating Characteristic): The Receiver Operating Characteristic curve shows the trade-off between Sensitivity and specificity.

The ROC curve is a graph with the following:

**The x-axis shows:**

Specificity: non-dangerous Asteroids anticipated as dangerous/ (non-dangerous Asteroids anticipated as hazardous +dangerous Asteroids anticipated as non-dangerous)

**The y-axis shows:**

Sensitivity: dangerous Asteroids anticipated as dangerous/ (dangerous Asteroids anticipated as dangerous+ non-dangerous Asteroids anticipated as non-dangerous)

**Micro-average**: the sum of the dangerous Asteroids correctly anticipated as dangerous/ the number of correctly non-dangerous Asteroids detected as non-dangerous.

**Macro-average**: (the sum of the dangerous Asteroids correctly anticipated as dangerous + the number of correctly non-dangerous Asteroids detected as non-dangerous)/2.

**Fig. 3:**
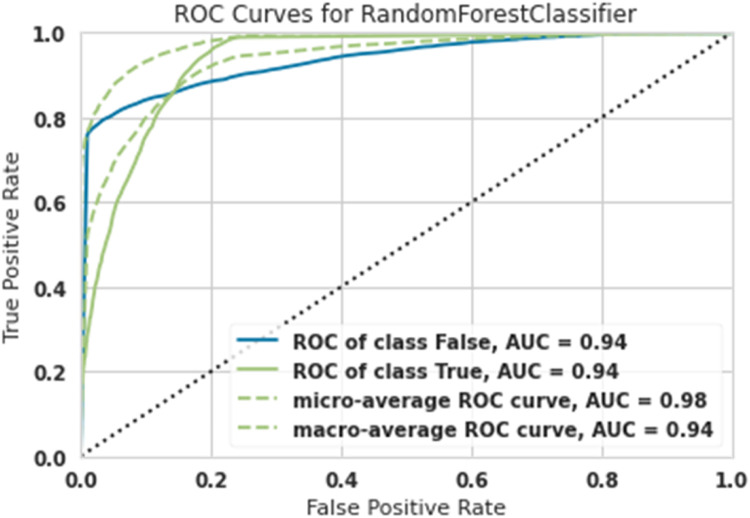
ROC Curves for Random Forest for the classification of hazardous objects (Asteroids).

**Fig. 4:**
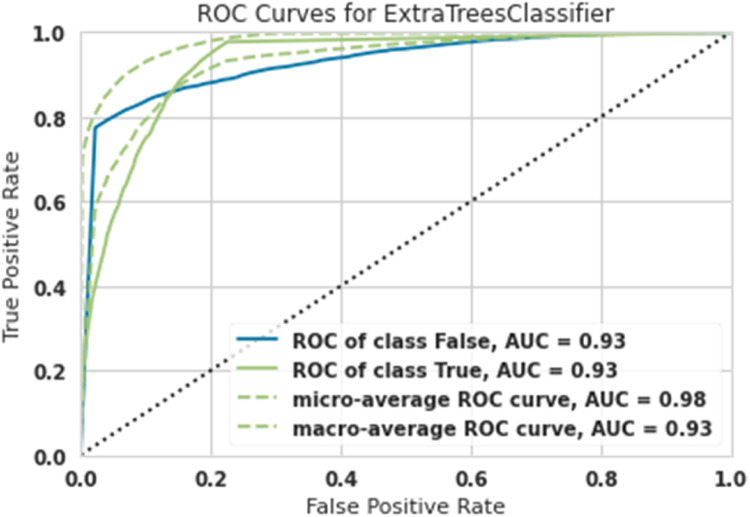
ROC Curves for Extra Tree for the classification of hazardous objects (Asteroids).

**Fig. 5:**
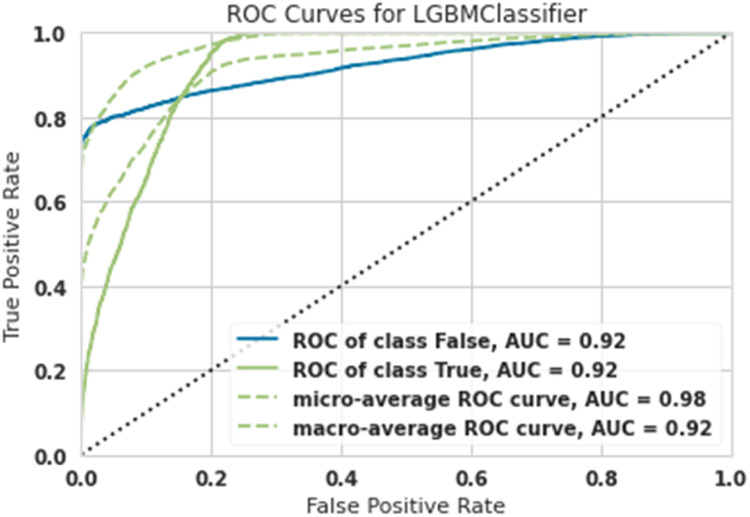
ROC Curves for Lightgbm for the classification of hazardous objects (Asteroids).

**Fig. 6:**
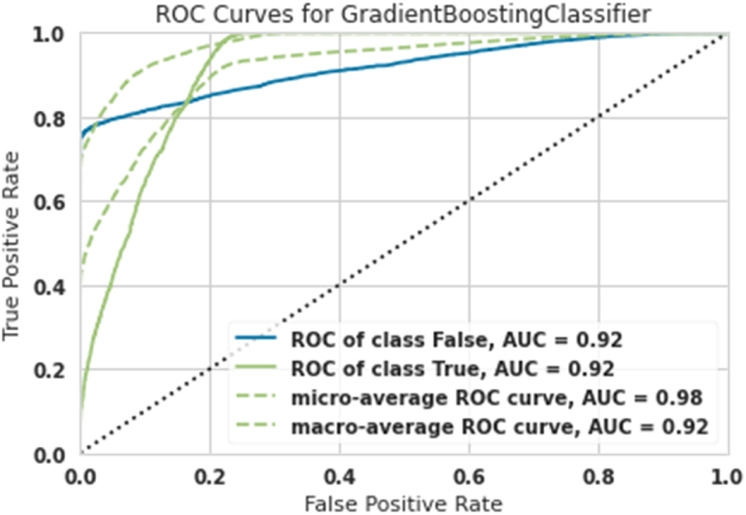
ROC Curves for Gradient Boosting for the classification of hazardous objects (Asteroids).

**Fig. 7:**
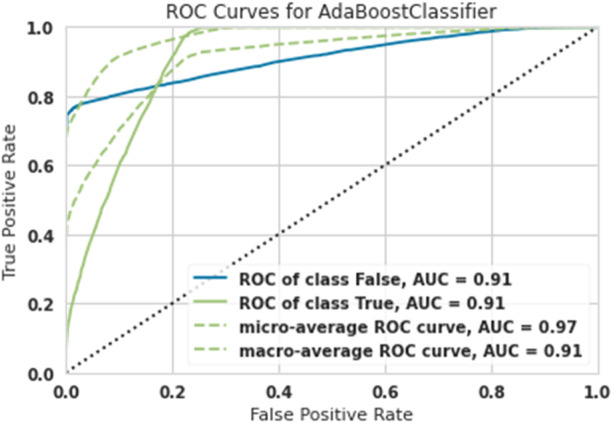
ROC Curves for Ada Boost for the classification of hazardous objects (Asteroids).

Table 1 shows the overall results with different training, test, and validation sizes and due to the best results belonging to Train-size=80%, Test-size=10% and Validation-size==10%, as shown in Fig. 3, Fig. 4, Fig. 5, Fig. 6, Fig. 7. As easily observed in this table, the results of Train-size=80%, Train-size=70% and Train-size=60% were not significantly different from each other.

**Table 1:** Performance of ML methods for classification of hazardous objects (Asteroids).

Train size:80 Test size:10 Validation size:10				
Method	ROC of class False	ROC of class True	Micro-average ROC curve	Macro-average ROC curve
Random Forest	0.94	0.94	0.98	0.94
Extra Tree	0.93	0.93	0.98	0.93
Lightgbm	0.92	0.92	0.98	0.92
Gradient Boosting	0.92	0.92	0.98	0.92
Ada Boost	0.91	0.91	0.97	0.91


Train size:70 Test size:15 Validation size:15				
Method	ROC of class False	ROC of class True	Micro-average ROC curve	Macro-average ROC curve
Random Forest	0.93.8	0.93.8	0.97	0.93
Extra Tree	0.92	0.92	0.97	0.92
Lightgbm	0.92	0.92	0.98	0.92
Gradient Boosting	0.912	0.912	0.975	0.91
Ada Boost	0.90	0.90	0.96	0.90


Train size:60 Test size:20 Validation size:20				
Method	ROC of class False	ROC of class True	Micro-average ROC curve	Macro-average ROC curve
Random Forest	0.93	0.93	0.968	0.938
Extra Tree	0.915	0.915	0.965	0.925
Lightgbm	0.92	0.92	0.98	0.92
Gradient Boosting	0.92	0.92	0.98	0.92
Ada Boost	0.90	0.90	0.96	0.90

The diagnostic capabilities of a binary classifier system when its discrimination threshold is changed are shown graphically by a ROC curve or receiver operating characteristic curve. While ROC (Receiver Operating Characteristic) stands for the probability curve, AUC (Area under the ROC Curve) stands for the amount of separability that is being measured. The model well represents class differences. The greater the AUC, the better the model correctly classifies true and false classes. Because the computations of micro-and macro averages (regardless of the measure) vary somewhat, their interpretations also change. Using a macro-average, the metric is computed separately for each class, and the average is then calculated (hence treating all categories equally).

A micro-average computes the average metric by combining the contributions of all classes. A micro-average in a multi-class classification system may detect the class imbalance.

In Fig. 3, the macro AUC curve is the lower green dashed line and the micro AUC curve is the upper green dashed line. Fig. 3 shows the ROC Curve for Random Forest; results show that the AUC was obtained for asteroids that did not pose a risk of 0.94, the AUC for asteroids with a risk of 0.94, and finally, the Micro-average was 0.98, and the macro-average was 0.94. This algorithm was the most powerful in the hazard classification of asteroids around the Earth. Both dangerous asteroids and non-dangerous asteroids were classified with 94% accuracy.

In Fig. 4, the macro AUC curve is the lower green dashed line and the micro AUC curve is the upper green dashed line Fig. 4 shows the ROC Curve for Extra Tree, and results show that the AUC was obtained for asteroids that did not pose a risk of 0.93, and the AUC for asteroids that had a risk was 0.93, finally, the Micro-average was 0.98, and the macro-average was 0.93. after the random forest. This algorithm was the most powerful in the hazard classification of asteroids around the Earth. Both dangerous asteroids and non-dangerous asteroids were classified with 93% accuracy.

In Fig. 5, the macro AUC curve is the lower green dashed line and the micro AUC curve is the upper green dashed line Fig. 5 shows the ROC Curve for Lightgbm; results show that the AUC was obtained for asteroids that did not pose a risk of 0.92, the AUC for asteroids that had a risk was 0.92, and finally, the Micro-average was 0.98, and the macro-average was 0.92. Both dangerous asteroids and non-dangerous asteroids were classified with 92% accuracy.

In Fig. 6, the macro AUC curve is the lower green dashed line and the micro AUC curve is the upper green dashed line Fig. 6 shows the ROC Curve for gradient boosting; results show that the AUC was obtained for asteroids that did not pose a risk of 0.92, the AUC for asteroids with risk was 0.92, and finally, the Micro-average was 0.98, and the macro-average was 0.92. Both dangerous asteroids and non-dangerous asteroids were classified with 92% accuracy.

In Fig. 7, the macro AUC curve is the lower green dashed line and the micro AUC curve is the upper green dashed line Fig. 7 shows the ROC Curve for Ada Boost; results show that the AUC was obtained for asteroids that did not pose a risk of 0.91, the AUC for asteroids that had a risk was 0.91, and finally, the Micro-average was 0.97, and the macro-average was 0.91. Both dangerous asteroids and non-dangerous asteroids were classified with 91% accuracy.

To detect the performance of machine learning algorithms in ROC chart classification and AUC, Micro-average, and Macro-average criteria, it correctly shows the algorithms' performance.

This article examined 90,836 asteroids at a distance of 70,000 km. High-risk and low-risk asteroids were classified using five algorithms: Lightgbm, Gradient Boosting, Ada Boost, Extra Tree, and Random Forest. The most robust performance was related to the Random Forest algorithm, and the weakest performance was related to the Ada Boost algorithm.

In such a way that the Random Forest algorithm predicted high-risk asteroids with 94% accuracy, and the Ada Boost algorithm predicted high-risk asteroids with 91% accuracy.

## Abbreviations

**AUC**:

The area under the ROC Curve

**CR**:

What does “**C**” mean in astronomy? It stands for the speed of light (c) times the redshift (z) as measured from the redshift of the spectrum. It is equal to the line-of-sight velocity that the star has. Positive means going away from us.

What does “**R**” stands for, astronomy? Radius. In astronomy, an instrument for measuring the angular distance between two celestial objects.

## Ethics statements

a) informed consent was obtained from participants
b) the platform(s)’ data redistribution policies were complied with method x journal

## CRediT authorship contribution statement

**Seyed Matin Malakouti:** Conceptualization, Methodology, Software, Validation, Data curation, Writing – original draft. **Mohammad Bagher Menhaj:** Visualization, Investigation, Supervision, Writing – review & editing. **Amir Abolfazl Suratgar:** Visualization, Investigation, Supervision, Writing – review & editing.

## Declaration of Competing Interest

The authors declare that they have no known competing financial interests or personal relationships that could have appeared to influence the work reported in this paper.

## Data Availability

Data will be made available on request.
